# The Price We Pay for the Convenience of Plastics

**DOI:** 10.1210/jendso/bvae022

**Published:** 2024-02-09

**Authors:** R Thomas Zoeller

**Affiliations:** Department of Biology, University of Massachusetts Amherst, Amherst, MA 01003, USA

**Keywords:** plastics, economic costs, endocrine disrupting chemicals, human health

A recent study published in the *Journal of the Endocrine Society* estimates the health care costs attributable to chemicals in plastics in the United States are on the order of $250 billion/year, or 1.22% of our gross domestic product [1]. Although this is a surprisingly large number, it is certainly a gross underestimation. Here are some of the reasons.

A trip to the grocery store is a good introduction to the prevalence of our nation's plastics use. Nearly all the plastics one sees will be in the form of single-use packaging, from laundry detergents to peanut butter to dairy products and individually wrapped fruits and vegetables. In fact, the largest market for plastics currently is packaging, which corresponds to a global shift to single-use containers [2]. These materials also break down into small particles in the micro- and nanoscale now found in human tissues, including placenta [3], fetal and infant feces, and breastmilk. A recent study even found that water bottled in plastic contains about 25 million plastic particles per liter [4]. But plastics are also prevalent in construction materials, medical devices, the automotive industry, and home and commercial appliances. Plastics are everywhere.

Plastics are not simply inert polymers but contain thousands of added chemicals that can leach into products and into people. The UN Environment Programme published a report in 2023 [5] concluding that there are more than 10 000 chemicals associated with plastics production with 10 classes identified as being hazardous to human health. These include specific flame retardants, certain UV stabilizers, per- and polyfluoroalkyl substances, phthalates, bisphenols, alkylphenols and alkylphenol ethoxylates, biocides, certain metals and metalloids, polycyclic aromatic hydrocarbons, and many other nonintentionally added substances.

To make their cost estimates, the Trasande group first analyzed the existing literature to identify plastic-related fractions of disease and disability for specific polybrominated diphenylethers (flame retardants), phthalates, bisphenols, and polyfluoroalkyl substances and perfluoroalkyl substances. They then updated previously published disease burden and cost estimates for exposure to these chemicals in the United States to 2018, based on biomonitoring data and epidemiological studies. By combining those data, they computed estimates of attributable disease burden and costs due to plastics in the United States.

Thus, Trasande et al used rigorous methods to estimate the disease burden for only a few chemicals used in plastic materials for which there are sufficient data and only a subset of diseases for those chemicals including IQ loss, male reproductive problems, diabetes and obesity, and cardiovascular disease. The clear implication is that their estimate of the health care costs related to plastics use in the United States is an underestimation of the total attributable costs.

The importance of the work by Trasande et al is that they provide an evidence-based estimate of the cost of our current system of plastics production, use, and end-of-use processes. We should recognize, however, that these estimates are not only an underestimate of the economic costs but also a surrogate measure of human suffering attributable to the chemicals in plastics. Therefore, “recycling” in the face of increasing production of new plastics will not reduce this human (and financial) cost. Only by reducing the production of new plastics can we hope to reduce the disease burden attributable to plastics through chemical exposures.

As Trasande et al point out, it was the economic loss associated with marine pollution that led to the United Nations Environment Assembly adopting a resolution to develop a global, legally binding plastics treaty. But in subsequent negotiations, the United States has objected to the concept of reducing plastics production [6]. The benefits of encasing reductions in plastics production are substantial because the concomitant reduction in chemical exposures will lead to savings in health care costs due to lower disease burdens. The benefits in the United States alone are likely to be in the billions of dollars annually. Moreover, the benefits in low- and middle-income countries where health care may be less available and plastic pollution is growing would be even greater in terms of human health.

If the economic benefits are not a sufficient argument for the United States to support agreements to reduce plastic production, the benefits to quality of life of all Americans should be.

## Disclosures

The author has nothing to disclose.
